# Supplementary material for: Study of the influencing factors of the liquid CO2 phase change fracturing effect in coal seams
Source: PLoS One. 2021 Jul 21;16(7):e0254996. doi: 10.1371/journal.pone.0254996 (PMC8294570; doi:10.1371/journal.pone.0254996)
Supplement: Attachment — Submitted filename: Response to Reviewers.docx [file pone.0254996.s001.docx]

Dear Editors and Reviewers:

Thank you for your letter and for the reviewers’ comments concerning our manuscript entitled “**Study of the influencing factors of the liquid CO_2_ phase change fracturing effect in coal seams**” (PONE-D-21-16377). Those comments are all valuable and very helpful for revising and improving our paper, as well as the important guiding significance to our researches. We have studied comments carefully and have made correction which we hope meet with approval. The main corrections in the paper and the responds to the reviewer’s comments are as follows:

**Response to Reviewer #1**

In this paper the current situation of low permeability gas extraction from coal seam gas difficult, will the influence factors of liquid CO_2_ phase change of coal seam crack effect as the research object, first analyzed theoretically, and through the simulation software to simulate the effects of different factors on the CO2 phase change crack damage effect, after using the mathematical algorithm to sort the factors important degree, finally carried out the field contrast test, This study provides a theoretical basis for the practical construction of liquid CO2 phase change cracking and anti-reflection technology in mines, and has some innovation. However, there are still some contents in the article that need to be modified. Suggestions are as follows:

1. In Section 5.1, the coal seam geological parameters should increase the raw coal gas content and the absolute gas emission amount at the working face.

**Response:** Considering the Reviewer’s suggestion, the author made the following changes: It has been added in the manuscript: the maximum raw coal gas content is 10.5 m^3^/t, the absolute gas emission from the working face is 18 m^3^/min,

2. Some of the formula sources in the paper need to add references. For example, Formula (10), because these formulas are not original to the author.

**Response:** Considering the Reviewer’s suggestion, the author has added corresponding references in the manuscript.

3. Can the liquid CO_2_ phase change induced cracking and anti-reflection technology described in this paper cause gas explosion?

**Response:** Liquid CO_2_ phase change blasting will not cause a gas explosion. Liquid CO_2_ phase change is a physical change, and no open flame will be generated during the blasting process. A large number of field verifications have shown that this technology is a relatively safe way to enhance the penetration of coal seams.

4. “The effective radius of fracture was measured to be approximately 357, 367, 381, and 396, and the coal body fracture degree was 6.35%, 6.56%, 6.81%, and 7.09%, respectively.” “The effective radius of fracture was measured to be approximately 357, 344, 331, and 322.” The parameters in these two sentences are missing units.

**Response:** Considering the Reviewer’s suggestion,the author has revised one by one in the manuscript.

5. Due to the length of the article, it is recommended that there should be no less than 30 references.

**Response:** Considering the Reviewer’s suggestion, the author has added appropriate literature to the manuscript.

6. The "R" and "M" on all simulation diagrams in this paper should be italicized because they are variables.

**Response:** Considering the Reviewer’s suggestion, the author has revised the pictures in the manuscript.

**Response to Reviewer #2**

Through the application of simulation software, this study analyzed the influence of physical parameters of coal seam on phase change cracking effect of liquid CO_2_, and verified the reliability of blasting numerical model through comparative engineering tests of different blasting parameters. The research results have a certain innovative significance for the application of liquid CO_2_ fracturing technology in coal mines. The study presents the results of original research. Results reported have not been published elsewhere. Experiments, statistics, and other analyses are performed to a high technical standard and are described in sufficient detail. Conclusions are presented in an appropriate fashion and are supported by the data. The article is presented in an intelligible fashion and is written in standard English. The research meets all applicable standards for the ethics of experimentation and research integrity. The article adheres to appropriate reporting guidelines and community standards for data availability.

**Response:** Thanks to reviewer for reviewing and supporting the manuscript.

**Response to Reviewer #3**

In this paper, based on the mechanical mechanism of coal rock crack propagation under the blasting load of liquid CO_2_ phase change, the influence of liquid CO_2_ phase change cracking effect was studied by means of simulation software, mathematical methods and field tests. The influence of thesis writing logic is stronger, system structure is reasonable, the writing more specification, research results have good practical value, and has certain innovative. However, this article still needs some simple changes. Suggestions are:

1. The difference and advantage of liquid carbon dioxide phase change anti-reflection technology compared with conventional charge blasting anti-reflection technology.

**Response:** The conventional explosives used in coal mines are explosives (TNT, RDX, etc.). During the blasting process, it is a chemical reaction. The blasting power is powerful, and it is easy to cause a large area of coal to be crushed, and it is easy to cause drilling during the blasting process. The gas in the hole is protruding, which has a certain degree of danger and is not easy to store. Liquid CO_2_ blasting is a physical reaction that uses high-pressure gas to crack the coal seam. There is no open flame during the blasting process, no gas explosion, and the peak pressure of blasting is between 150-300MPa, and it will not cause large-scale damage to the coal seam. The technology is simple to operate, convenient to store, and is widely used in the mining field.

2. The "blasting hole" in Fig.13 and the "fissionable hole" in Table 5 describe a thing, so please be consistent and ensure that the whole article is consistent.

**Response:** Considering the Reviewer’s suggestion, the author has revised the manuscript.

3. Will there be secondary pollution after SF_6_ gas is injected into coal seam?

**Response:** SF_6_ is a colorless, odorless, inert, non-combustible gas. This gas is insoluble in water, has no sedimentation, does not condense, and is not adsorbed by the surface of downhole materials. It is a good negative gas. The detection sensitivity of SF6 is high, and it can be effectively detected by gas chromatograph with electronic trap or SF6 detector. It can be conveniently and accurately applied to the measurement of the effective radius of coal seam boreholes. It is the most ideal tracer gas.

4. Some formulas in the literature need to be quoted. Variables in the formulas need to be represented in italics and constants need to be represented in upright letters. Please check the full text to ensure accuracy.

**Response:** Considering the Reviewer’s suggestion, the author has revised the manuscript.

**Response to Reviewer #4**

This is an interesting paper that contains some valuable new data about the study the liquid CO_2_ phase change fracturing of coal seam. It also analyzes various influencing factors and their contributions. This has certain guiding function to theory and practice. Still, the major modifications or clarifications should be made.
Detailed comments
1. It will be more appropriate to change the statement of "grey correlation comprehensive index" to "grey correlation cumulative index".

**Response:** Considering the Reviewer’s suggestion, the author has revised the manuscript.

2. Some mathematical symbols in the formula, if the value is fixed, can not use italics, please correct the English punctuation marks.

**Response:** Considering the Reviewer’s suggestion, the author carefully proofreads and has made revisions in the manuscript.

3. “2.2.5 Mechanism of coal rock crack extension under liquid CO_2_ phase change airburst loading”- This part is based on the linear elastic fracture mechanics explains the mechanism of crack extension of the coal. However, the coal is actually not a brittle material, it is a quasi brittle materials. There will be a fracture process zone (FPZ) at the crack tip of a quasi-brittle material. Due to its existence, the linear elastic fracture mechanics is no longer applicable. The influence of FPZ on the fracture process is significant in the static process, and has been extensively studied, but the research in the dynamic process is still insufficient. The author can refer to related references for further explanation. This will be helpful for in-depth understanding of coal fracture process. If possible, it is suggested to increase the effect of fracture process zone on the fracturing effect.

**Response:** Based on the suggestions of reviewers, the author learned that coal is a quasi-brittle material, and linear elastic fracture mechanics is no longer applicable, which is of great help to the author. The author has made revisions in the manuscript as suggested. Regarding the fracture process zone (FPZ) problem raised by experts, the author thinks this is a good research direction, but because the author focuses on the study of the influence of different factors on the blasting effect of liquid CO_2_, the problem has not been analyzed and explained in detail.The author will do further in-depth research in the future research, and hope that the expert will understand.

4. The coal seam parameter description in Chapter 5 is a little less, so some other parameters should be added appropriately.

**Response:** Considering the Reviewer’s suggestion, the author has added in the manuscript: the maximum raw coal gas content is 10.5 m^3^/t, and the absolute gas emission from the working face is 18 m^3^/min.

**Response to Reviewer #5**

This paper studies the effects of different physical parameters (in situ stress, gas pressure, modulus of elasticity and strength of coal) and blasting parameters (fracturing pore size and peak pressure of detonation ) on the phase change fracturing effect of liquid CO_2_. After reading, I think there are some problems in the structure, format and logic of the manuscript. I suggest that it be revised and published. It mainly includes the following aspects:

1. There are no keywords in the text.

**Response:** Considering the Reviewer’s suggestion, this article has added corresponding keywords in the manuscript, as follows:

Keywords: liquid CO_2_ phase change fracturing; influencing factors of the cracking effect; blasting numerical model; graygrey correlation theory; engineering tests

2. Some paragraphs of the whole article are malformed, without indenting the first line.

**Response:** Considering the Reviewer’s suggestion, the author has revised the manuscript in conjunction with the journal template.

3. In Section 2.1, title format is wrong.

**Response:** Considering the Reviewer’s suggestion, the author has revised the manuscript in conjunction with the journal template.

4. The title of the chart is not centered and the formula label is not aligned.

**Response:** Considering the Reviewer’s suggestion, the author revised the wrong format.

5. Section 3 does not specify the specific simulation software. What is the reference basis for building the model?

**Response:** Considering the Reviewer’s suggestion, this paper uses ANSYS/LS-DYNA software, which has been explained in the manuscript.

The basis of modeling is based on the actual conditions of the test working face of the Mabao coal mine and combined with the relevant coal and rock parameters in the literature[22,23]

6. The picture is not clear.

**Response:** Considering the Reviewer’s suggestion, the author has reprocessed the image and used plos one's (PACE) digital diagnostic tool for proofreading

7. There are many paragraphs in the conclusion, which are suggested to be explained in sections.

**Response:** Considering the Reviewer’s suggestion, the author has explained the conclusion part by chapter in the manuscript.
